# The metabolic phenotype of rodent sepsis: cause for concern?

**DOI:** 10.1186/2197-425X-1-6

**Published:** 2013-10-29

**Authors:** Parjam S Zolfaghari, Bernardo Bollen Pinto, Alex Dyson, Mervyn Singer

**Affiliations:** Bloomsbury Institute of Intensive Care Medicine, University College London, Cruciform Building, Gower St, London, WC1E 6BT UK; Department of Anaesthesiology, Intensive Care and Emergency, Santo António Hospital, Largo Professor Abel Salazar, Porto, 4099-001 Portugal; Graduate Program in the Areas of Basic and Applied Biology, University of Porto, Rua Dr. Roberto Frias, 4200-465 Porto, Portugal

**Keywords:** Faecal peritonitis, Metabolism, Oxygen consumption, Haemodynamics

## Abstract

**Purpose:**

Rodent models of sepsis are frequently used to investigate pathophysiological mechanisms and to evaluate putative therapeutic strategies. However, preclinical efficacy in these models has failed to translate to the clinical setting. We thus questioned the representativeness of such models and herein report a detailed comparison of the metabolic and cardiovascular phenotypes of long-term faecal peritonitis in fluid-resuscitated rats and mice with similar mortality profiles.

**Methods:**

We conducted prospective laboratory-controlled studies in adult male Wistar rats and C57 black mice. Animals were made septic by intraperitoneal injection of faecal slurry. Rats received continuous intravenous fluid resuscitation, whereas mice received intermittent fluid boluses subcutaneously. Sham-treated animals served as controls. Survival was assessed over 72 h. In separate studies, whole body metabolism (O_2_ consumption, CO_2_ production) was measured over 24 h with echocardiography performed at early (6 h) and established (24 h) phases of sepsis. Blood gas analysis was performed at 6 h (rats) and 24 h (rats, mice).

**Results:**

Similar survival curves were seen in both rodent models with approximately 75% mortality at 72 h. In mice, sepsis caused severity-dependent falls in core temperature and global metabolism. Oxygen consumption in severely septic mice fell by 38% within 2 h, and 80% at 22 h compared with baseline values. This was only partially restored by external warming. By contrast, septic rats maintained core temperature; only severely affected animals showed a pre-mortem decline in oxygen consumption. Significant myocardial dysfunction was seen in mice during early and established sepsis, whereas peak velocity and other hemodynamic variables in rats were similar at 6 h and significantly worse by 24 h in severely septic animals only.

**Conclusions:**

Markedly differing metabolic and cardiovascular profiles were seen in long-term fluid-resuscitated rat and mouse models of bacterial sepsis despite similar mortality. The mouse model, in particular, does not represent the human condition. We urge caution in applying findings in murine models to septic patients, both with regard to our understanding of pathophysiology and the failure to translate preclinical efficacy into successful clinical trials.

**Electronic supplementary material:**

The online version of this article (doi:10.1186/2197-425X-1-6) contains supplementary material, which is available to authorized users.

## Introduction

Severe sepsis is the exaggerated host inflammatory response to an infectious insult leading to multiple organ failure (MOF) and, in many cases, death [1]. The underlying pathophysiology remains poorly understood, and targeted treatments that improve outcomes remain elusive. Rodent models are routinely used to investigate both pathophysiological mechanisms and novel therapeutic strategies. This is facilitated by rapid breeding, ease of care, relatively low costs and powerful genetic technology [2]. A PubMed search using the words ‘endotoxin’ or ‘sepsis’ for ‘mouse’ and ‘rat’ yielded 10,500 and 5,100 citations, respectively. However, numerous positive therapeutic intervention studies in such laboratory models have failed to translate into clinical benefit in subsequent human trials. This has resulted in much closer scrutiny of these models and their relevance to patients [3, 4]. Many are poorly representative of the human condition in terms of the type of septic insult (e.g. injection of endotoxin), study duration (hours rather than days), lack of any resuscitation (such as fluid), and the timing of putative therapies (often given at or preceding the insult rather than after establishment of organ dysfunction, as would be the case in patients). The physiological, immunological and biochemical phenotype of these models, and temporal changes thereof, are also largely unknown.

Over the last decade, we have attempted to create well-characterized rat and mouse models that are more representative of the human condition in terms of type (faecal peritonitis) and duration of insult (≥24 h), with fluid resuscitation to avoid tissue hypoperfusion related to untreated hypovolaemia [5–7]. Here, we report a detailed assessment of the cardiovascular, metabolic and biochemical phenotypes in long-term rat and mouse sepsis models with similar mortality. Concurrent with a recent publication reporting non-concordance in leukocyte transcriptional changes between human and murine systemic inflammatory states including sepsis [8], we describe an early severity-related hypometabolic and hypodynamic state in our mouse model that further calls into question its validity as a paradigm for human sepsis.

## Materials and methods

All experiments were performed in accordance with the UK Animals (Scientific Procedures) Act of 1986 and approval from the University College London Ethics Committee. All animals were housed under standard conditions with water and chow diet *ad libitum*. Operative procedures and echocardiography were performed under general anaesthesia while the animals were breathing spontaneously through a facemask entrained with a mixture of room air and isoflurane. Animals were externally warmed during anaesthesia to maintain body temperature between 36°C and 38°C. In mice, the depth of anaesthesia was titrated at each time point using the loss of reflex withdrawal to pain as the end point to minimize the cardiovascular effects of isoflurane. In rats, a fixed percentage (2.5% during surgical procedures and 1.5% for induction of sepsis and echocardiography) of isoflurane was used throughout. Room temperature was maintained at a constant 21°C.

### Rat model

Wistar rats (male, with approximately 300 g body weight, 12 to 14 weeks old) were anaesthetized and placed in a supine position. Following skin preparation, the left common carotid artery and right internal jugular vein were exposed and cannulated using PVC tubing with 0.96-mm outside diameter (Biocorp Ltd, Huntingdale, Australia). These were tunnelled subcutaneously to the back of the neck and attached to a swivel/tether system (InsTech, Plymouth Meeting, PA, USA). The neck incision was closed, and subcutaneous (s/c) buprenorphine (0.05 mg/kg) (Vetergesic, Reckitt Benckiser, York, UK) was administered for pain relief prior to cessation of anaesthesia. The tether system allowed free movement around the cage with *ad libitum* access to food and water. The arterial line was connected to a pressure transducer (Powerlab, AD Instruments, Chalgrove, Oxon, UK) for continuous measurement of mean arterial pressure recorded using a 16-channel Powerlab system and Chart 5.0 acquisition software (AD Instruments, Chalgrove, Oxon, UK).

Twenty-four hours after line insertion, sepsis was induced with an intraperitoneal injection of faecal slurry (3 ml/kg body weight) administered through a 19G needle inserted into the right lower quadrant of the abdomen with care taken to avoid bowel perforation. Sham animals (control group) received an equivalent volume of 0.9% saline. Standardized human slurry (pooled stool from three healthy, non-vegetarian donors) was kindly donated by the Department of Anesthesiology and Intensive Care at Friedrich Schiller University, Jena, Germany [9]. A continuous infusion of 6% hydroxyethyl starch 130/0.4 (Volulyte, Fresenius Kabi, Bad Homburg, Germany) and glucose (1:1) at 10 ml/kg/h was administered through the internal jugular line commencing 2 h post-induction of sepsis. For survival studies exceeding 24 h, the rate was halved at 24-h intervals. These studies were performed prior to knowledge of the negative outcomes seen with starch-based intravenous fluids [10, 11]. Pilot studies revealed significant hypoglycaemia if glucose was not administered.

### Mouse model

C57 black mice (male, 25 to 35 g body weight, 18 to 30 weeks old) were initially made septic using the same technique as for the rats, with prior carotid and internal jugular cannulation and connection to a tether system. However, this method induced significant morbidity and mortality in the sham-operated group so was abandoned in favour of a simplified model without invasive arterial and venous cannulae. Sepsis was induced by injection of faecal slurry (20 ml/kg body weight) into the peritoneal cavity through a small (4 mm) transverse abdominal skin incision. Sham animals received the same incision and were injected with an equivalent volume of 0.9% saline. Faecal slurry was made fresh each day by diluting caecal content of rats (1:7 dilution with 0.9% saline). Mice were given 10 ml/kg of 0.9% saline s/c at the end of the procedure and received further s/c boluses of 50 ml/kg of pre-warmed 5% glucose/0.81% saline at 6 and 12 h thereafter for both fluid resuscitation and prevention of hypoglycaemia.

### Basic measurements and biochemistry

A clinical scoring system (Table 1) was used to record the severity of sepsis for each animal. This scoring system was previously validated in rats [5]. Clinical scores of 0 to 3 implied mild sepsis and ≥4 severe sepsis. Rectal temperature was measured at 0-, 6-, 18- and 24-h time points. At 24 h post-insult, mice were rapidly anaesthetized and the blood obtained by cardiac puncture as a terminal event. In rats, the blood was drawn from the arterial line without using anaesthesia. Samples were analyzed for blood gases and biochemistry.

**Table 1:** **Clinical severity score characteristics**

Characteristic	Scoring range
Hunched	0-1
Bloated	0-1
Conjunctival injection/mucky eyes	0-1
Piloerection (rats only)	0-1
Lack of movement	0-2
Lack of alertness	0-2

Scoring denotes absence (0), presence (1) or marked presence (2). Total score of 0 to 3 denotes mild sepsis and ≥4 severe sepsis.

### Metabolic rate

Following induction of sepsis and in-time-matched sham-operated controls (23 rats and 19 mice in total), whole body metabolic rate was measured for 24 h in individual metabolic chambers (Oxymax System, Columbus Instruments, Columbus, OH, USA). Gas samples from each box were sampled for 90 s at 8-min intervals. Oxygen consumption (VO_2_) and CO_2_ production (VCO_2_) were calculated using standard formulae. The respiratory exchange ratio (RER) was calculated as the ratio of VCO_2_ to VO_2_. Values near 1 indicate a predominance of carbohydrate metabolism while values approaching 0.7 indicate fatty acid oxidation [12].

Animals were acclimatized in their metabolic cages for at least 3 h prior to induction of sepsis. All experiments were started at the same time of day to avoid bias due to diurnal variation in metabolic rate. Mice were recovered for 1 h in a warm chamber following general anaesthesia prior to returning to their cages.

In separate studies, mice were removed from their metabolic cages at 10 and 24 h and then re-warmed in a heating chamber to 37°C (rectal temperature) over 1.5 h. Echocardiography was performed under a brief period of isoflurane anaesthesia before and immediately after re-warming, and then the mice were returned promptly to their metabolic cages where oxygen consumption recordings were resumed within 1 to 2 min.

### Cardiac output measurement

Echocardiography was performed at 0, 6 and 24 h in sham and septic animals using a 14-MHz probe connected to a Vivid 7 Dimension echocardiography machine (GE Healthcare, Chalfont St. Giles, Bucks, UK). The aortic blood flow velocity was measured in the proximal ascending aorta immediately before the bifurcation of the right carotid artery using pulse-wave Doppler. Aortic diameters in rats and mice of this age are, respectively, 2.6 mm [13] and 1.35 mm (S Hollenberg, personal communication). Stroke volume was calculated by multiplying the velocity time integral (VTI) from six consecutive cycles (equivalent to one respiratory cycle) by the aortic cross-sectional area. The average peak-to-peak distance and maximum velocity over the six consecutive systolic cycles were used to measure heart rate and peak velocity, with the latter being a marker of left ventricular contractility [14]. Cardiac output was calculated as the product of stroke volume and heart rate.

### Survival studies

In separate studies, animals were followed for up to 72 h to assess survival.

### Statistics

PASW Version 18.0 (SPSS Inc., Chicago, IL, USA) was used to carry out statistical tests. All variables were tested for normality of distribution using the Kolmogorov-Smirnov test. All parametric data were compared using Student’s *t* test or analysis of variance (one-way or two-way with repeated measures). Statistical significance level was set at *p* < 0.05. Tukey’s HSD and Dunnett’s *post hoc* tests were used to ascertain the significance between groups.

## Results

**Figure 1:**
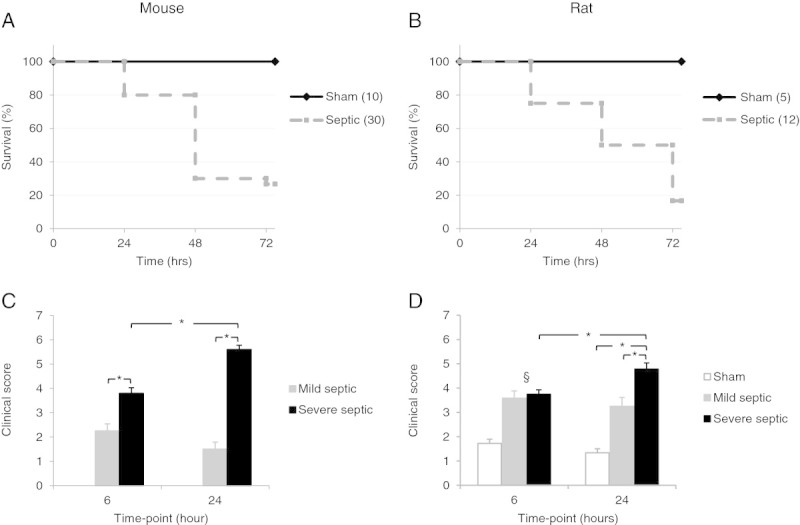
**Seventy-two hour survival curves in the mouse (A) and rat (B) models of sepsis.** Clinical severity was scored for each animal at 6 and 24 h using a previously published scoring system (5). The average score (± SEM) for mildly **(C)** and severely septic **(D)** animals is shown. Sham mice scored 0 at both time points. *p < 0.01 one-way ANOVA and §*p* < 0.01 (ANOVA), comparing mild and severe to the sham group in rats at 6 h.

Survival studies were carried out in 10 sham and 30 septic mice, and 5 sham and 12 septic rats. Similar survival curves were observed in both rodent models; most of the deaths had occurred by 48 h with an approximately 75% mortality at 72 h (Figure 1A,B). Animals surviving to this point showed signs of clinical recovery. Of the septic animals surviving to 24 h, 38% of the mice were scored as mild and 62% as severe at this time point, while 33% of the rats were scored mild and 66% severe. The clinical score at 6 and 24 h in the mice and at 24 h in the rats is related to subsequent mortality (Figure 1C,D).

**Figure 2:**
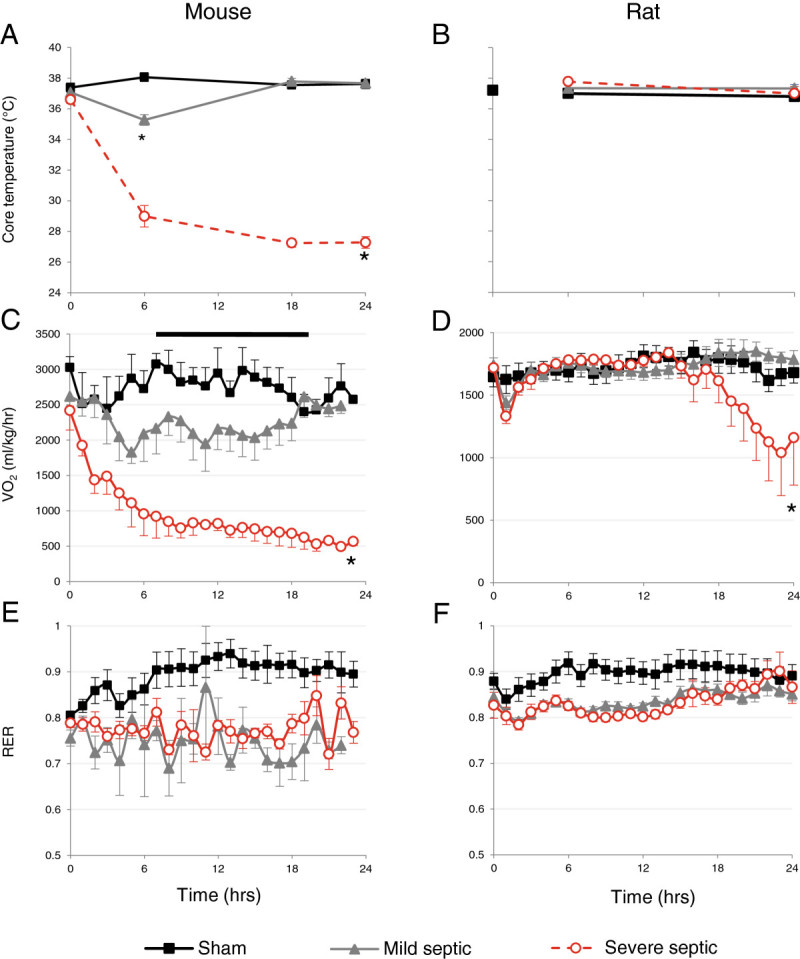
**Core temperature (A,B), VO**_**2**_ **(C,D) and RER (E,F) of sham, mildly and severely septic mice and rats.** Results are mean ± SEM. *p < 0.01 two-way ANOVA.

Metabolic measurements in mice were made over 24 h in six controls, six with mild sepsis and seven with severe sepsis. Sham-operated control mice remained normothermic and showed normal diurnal rhythmicity in VO_2_, both consistent with an increase in nocturnal activity (Figure 2A,C). By contrast, all septic mice became hypothermic with a significant severity-related drop in temperature at 6 h (*p* = 0.001 two-way ANOVA). However, while core temperature fell by 1.5°C in mildly septic mice, severely septic animals became profoundly hypothermic, reaching approximately 29°C by 6 h and failing to recover (Figure 2A). The core temperature fall in mice mirrored severity-dependent reductions in VO_2_ and VCO_2_ (Figure 2C). This was associated with a complete absence of variability (diurnal and movement/feeding related) in the individual VO_2_ traces. Compared to baseline values, VO_2_ in the severely septic mice fell by 38% ± 9% within 2 h, by 63% ± 8% within 6 h and as high as 80% ± 3% reduction at 22 h. In contrast, VO_2_ in mildly affected mice fell by 30% ± 7% at 6 h. Whereas no recovery was seen in severely septic animals, core temperature and metabolic rate recovered to normal values in the mildly septic mice by 18 h, in line with their clinical improvement.

**Figure 3:**
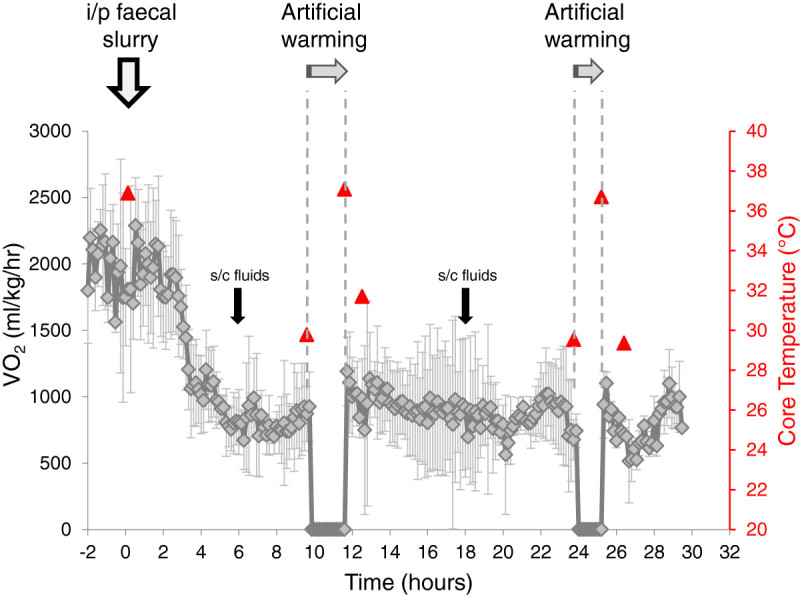
**Change in metabolic rate on rewarming.** VO_2_ and core temperature of mice with severe sepsis undergoing two episodes of rewarming over a 1.5 h time period (marked by the dashed lines). Mice were treated with s/c fluid boluses at 6- and 18-h time points (error bars denote standard deviation). *p < 0.05.

To assess the effects of hypothermia, separate re-warming studies showed partial restoration of haemodynamics at 10 h and complete restoration at 24 h; however, oxygen consumption only increased from 32% to 40% of the baseline values to 41% to 52% and quickly fell thereafter (Table 2, Figure 3).

**Table 2:** **Temperature, metabolic and echocardiography data in severely septic mice**

	Baseline	10-h		24-h	
		Pre-rewarming	Post-rewarming	Pre-rewarming	Post-rewarming
Temperature (°C)	36.1 ± 0.2	30.0 ± 1.5*	36.3 ± 0.6**	28.8 ± 2.1*	36.6 ± 1.7
VO_2_ (ml/kg/h)	2,288 ± 181	925 ± 131*	1,190 ± 131**	730 ± 90*	941 ± 178
Peak velocity (m/s)	1.06 ± 0.06	0.46 ± 0.08*	0.80 ± 0.10**	0.66 ± 0.07*	1.14 ± 0.13
Heart rate (beats/min)	509 ± 31	452 ± 23	601 ± 45**	435 ± 6	614 ± 47
Stroke volume (μl)	60 ± 3	21 ± 3*	32 ± 6**	36 ± 4*	52 ± 11

Data were taken from severely septic mice at baseline (0 h) and before and after rewarming at 10 h (6 mice) and 24 h (3 mice). Rewarming took place over 1.5 h. Mice were only anaesthetized for the period of echocardiography. Values denote mean ± standard deviation. *p < 0.05 paired *t* test comparing the values to corresponding baseline (0 h) values. **p < 0.05 paired *t* test comparing post-warming values to their corresponding pre-warming value.

In stark contrast to the early metabolic phenotype observed in mice, septic rats maintained their core temperature (Figure 2B). While a transient 15% ± 2% fall in VO_2_ (*p* = 0.001) was observed in the first hour following slurry administration in both mildly (*n* = 6) and severely affected (*n* = 7) rats, this promptly recovered to sham values (Figure 2D). Despite maintaining their core temperature, the severely septic rats showed a progressive decline in VO_2_ from 18 h onwards, in tandem with their clinical deterioration. Three of the severely affected rats died by 24 h, whereas all the mildly septic rats survived the 24 h duration of the study.

The RER of sham mice exceeded 0.9 from 8 h onwards, whereas in both mild and severe sepsis this fell promptly to approximately 0.75 (Figure 2E). Food intake was markedly decreased in the septic animals. A similar difference in RER was seen in the sham and septic rats, albeit with recovery towards sham values in both mildly and severely affected septic subsets (Figure 2F).

**Figure 4:**
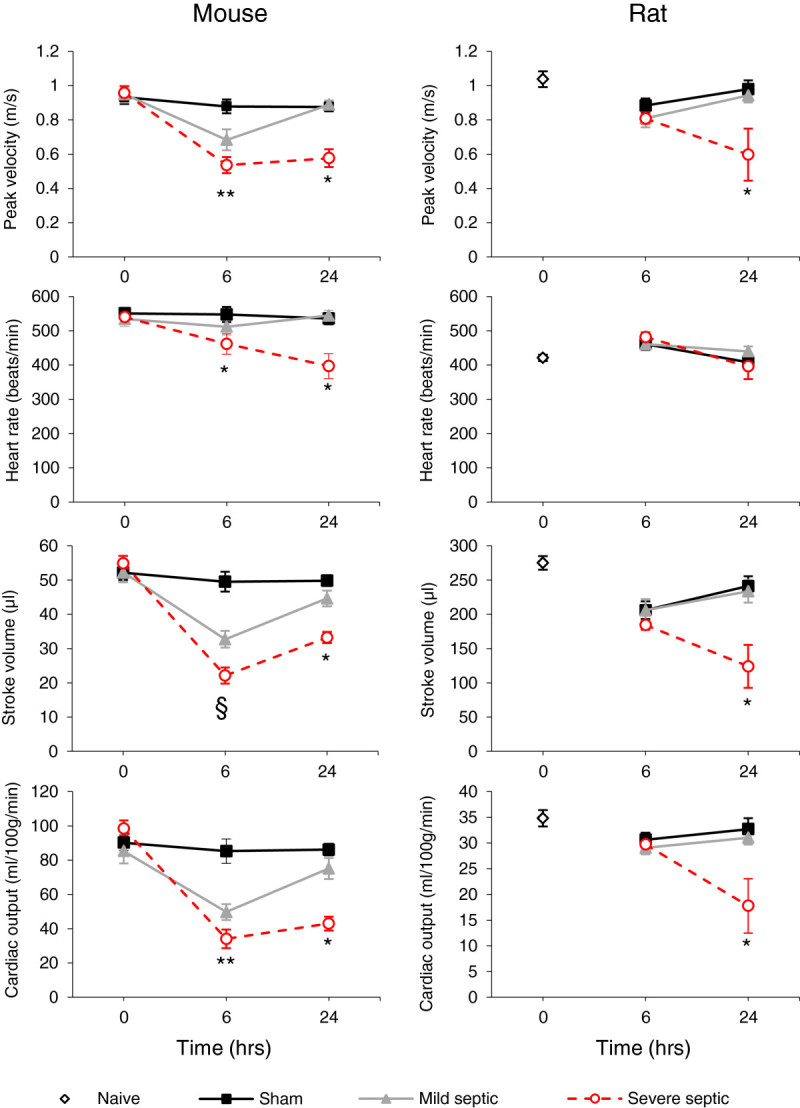
**Echocardiography results at baseline, 6 and 24 h after induction of sepsis in mice and rats.** *p < 0.05 significance severely septic group against other groups; ** *p* < 0.05 significance both mildly and severely septic against sham; § *p* < 0.05 significant difference between all groups (all two-way ANOVA).

Echocardiography was performed in 10 sham and 30 septic mice under isoflurane anaesthesia. Baseline echocardiographic variables were similar in both sham and septic mouse groups (Figure 4). However, at 6 h, all measured variables were significantly lower in the septic mice (*p* < 0.05). By 24 h these had recovered in the mildly septic mice but showed no improvement in severely affected animals.

Five naïve, 17 sham and 25 septic rats underwent echocardiography. In contrast to the mice, peak velocity and other haemodynamic variables were similar in septic and sham animals at 6 h and unchanged from baseline values in naïve rats. However, these measures significantly deteriorated by 24 h in the severely septic animals (Figure 4).

Blood sampling in mice at 24 h revealed no hypoxaemia but a marked mixed metabolic and respiratory acidosis in the severely septic mice (Table 3). Acid–base balance and arterial partial pressure of oxygen were unaffected in the rats, even in the severely septic group where mild hypocapnia was observed. However, both species became hyperlactataemic. Despite the aggressive fluid resuscitation, both septic mice and severely septic rats showed significant haemoconcentration. Due to administration of glucose, blood sugar levels were well maintained in both species with no recorded episodes of hypoglycaemia.

**Table 3:** **Blood gas results (mean ± SEM) at 24 h in sham, mildly septic (rats only) and severely septic mice and rats**

	Mouse		Rat		
	Sham	Severe septic	Sham	Mild septic	Severe septic
pH	7.33 ± 0.03	6.96 ± 0.12*	7.45 ± 0.02	7.44 ± 0.02	7.52 ± 0.04
PCO_2_ (kPa)	4.29 ± 0.29	8.46 ± 4.14*	4.3 ± 0.1	4.4 ± 0.1	3.2 ± 0.2*
PO_2_ (kPa)	13.4 ± 4.2	12.2 ± 4.3	13.8 ± 0.2	14.2 ± 0.1	17.6 ± 2.6
HCO_3_^−^ (mmol/l)	17.7 ± 0.5	10.9 ± 1.7*	23.9 ± 1.7	23.7 ± 0.8	25.0 ± 1.6
Cl^−^ (mmol/l)	122 ± 5	122 ± 2	106 ± 2	106 ± 2	104 ± 2
BE (mmol/l)	−8.5 ± 0.7	−18.0 ± 3.2*	−1.3 ± 1.9	−1.3 ± 0.9	−3.0 ± 2.6
Lactate (mmol/l)	2.4 ± 0.9	5.0 ± 1.2*	0.8 ± 0.1	1.7 ± 0.3	2.6 ± 0.4*
Glucose (mmol/l)	11.7 ± 4.0	14.9 ± 2.4	7.0 ± 0.7	6.4 ± 0.8	5.4 ± 0.7
Hb (g/dl)	13.2 ± 0.2	15.1 ± 1.6*	12.2 ± 0.5	12.0 ± 1.0	13.9 ± 0.9
MAP at 24 h (mmHg)	-	-	118 ± 2	100 ± 10	95 ± 10

MAP, mean arterial pressure; Hb, haemoglobin concentration; BE, base excess. *p < 0.05 comparing severely septic to sham and mildly septic animals.

## Discussion

The phenotypic differences between septic rodents and humans are poorly characterized. In this study we describe profound differences in metabolic and cardiovascular changes in severe, long-term models of rat and murine sepsis. Although the origin of the faecal slurry and the route of fluid administration differed, the age of the animals (approximating to juvenile/early adulthood) and laboratory conditions were similar, as were the time to death and the overall 72-h mortality rate (approximately 75%) of both models.

The major finding of our study was the marked and rapid decrease in metabolism in septic mice. While significant hypothermia is recognized following endotoxin or bacterial insults in mice [15–20], the 80% reduction in metabolic rate has not, to our knowledge, been previously reported. There was a corresponding change in cardiovascular phenotype with rapid severity-dependent reductions in heart rate and stroke volume. Despite an almost twofold rise in the cardiac output (and hence oxygen delivery) following re-warming of the septic mice, there was only a very modest rise in VO_2_. This implies that the metabolic suppression in the septic mice is independent of the core temperature or oxygen delivery. By contrast, the septic rats did not exhibit early falls in metabolic rate nor did they become hypermetabolic or hyperthermic. Falls in VO_2_ were seen over the 4 to 6-h period pre-mortem.

In humans the early response to low-dose endotoxin injection in volunteers is a hypermetabolic response with 89% and 46% rises in oxygen delivery and consumption, respectively by 3 h [21]. However, in septic patients, the increased metabolic response is progressively blunted with increasing severity such that the resting energy expenditure in was equivalent to normal healthy values [22]. Only in the recovery phase was a hypermetabolic response (up to 60% increase) noted [22, 23]. By contrast, and as we found with the septic rats, VO_2_ fell in critically ill patients during their dying process [24]. The reduced respiratory exchange ratio in the septic mice implies predominance of fatty acid oxidation as compared to the sham animals that use carbohydrate as their main energy source [12], even though high lactate levels result in an increase in VCO_2_ and RER [25]. This switch in fuel utilization is often seen in sick animals and during starvation and has also been reported in septic mice [20]. Studies in healthy mice starved for 20 h confirmed a similar fall in RER to 0.73 with a 25% fall in VO_2_ that reversed quickly on re-provision of food (data not shown). Notably, temperature did not change in this starvation model. Furthermore, as there was only an 11% variation in the VO_2_ observed with sleep-wake cycles in naïve mice (Additional file 1: Figure S1), decreased activity in the septic mice is likely to make only a relatively minor contribution to the profound reduction in metabolic rate observed.

The different metabolic responses within the two rodent species may reflect the presence of intact hibernatory pathways in mice. Metabolic suppression has been described in various organisms including mice under unfavourable situations such as cold, food or fluid deprivation, and hypoxia [26]. This strategy of ‘cross-tolerance’ results in a balanced reduction in energy production and utilization [27], enabling continuation of life-dependent processes such as membrane pumps at the expense of other energy-dependent functions such as protein synthesis [28, 29]. Metabolic suppression may be also triggered by a reduction in energy supply [30, 31]. Hibernation has been described in the septic mouse heart [32] and suggested in human critical illness [33, 34]. Metabolic suppression may be an adaptive response in severe sepsis in order to conserve energy [34] and, perhaps, limit reactive species production.

Mice have a very high metabolic rate (VO_2_ of 60 to 80 ml/kg/min compared with 3 to 4 ml/kg/min in humans), largely due to non-shivering thermogenesis to maintain body temperature [35]. It is difficult to assess the contribution of loss of non-shivering thermogenesis to the reduction in VO_2_ and core temperature observed in our mouse model. While it is tempting to speculate that this may occur, our study was designed to measure total oxygen consumption and could not distinguish between coupled and uncoupled respiration. Non-shivering thermogenesis is mediated by mitochondrial uncoupling proteins (principally UCP-1 in brown fat) as a result of beta-adrenergic stimulation [36]. Interestingly however, both catecholamine levels and UCP protein levels rise in sepsis [37–40]. The significance of upregulation of the uncoupling proteins UCP-2 and UCP-3 in sepsis is still unclear; however, there is no evidence that these influence thermoregulation [39].

Our protocol utilized fluid resuscitation, equivalent to 150 ml/kg s/c for mice and 220 ml/kg i/v in rats over the first 24 h. Notwithstanding these large volumes that are considerably higher than those reported in the literature [4], haemoglobin concentrations still rose at 24 h in both severely septic rats and mice compared to sham animals, implying haemoconcentration due to increased capillary leak. While hypovolaemia may have contributed in part to the reduced cardiac output seen at 6 and 24 h in mice and at 24 h in rats, the lack of tachycardia does imply that intrinsic sepsis-related myocardial depression and/or hypothermia (in the mice) also play a significant role. Transient re-warming of the mice to 37°C increased cardiac output (fully at 24 h) but only had a partial effect on global oxygen consumption. This was contrary to the previous observations by Rudaya et al. [18] who noted that thermoregulatory responses in LPS-treated mice were dependent on the ambient temperature. Our data imply that metabolic shutdown is an important phenotypic response to sepsis in mice.

Severity as measured by the clinical scoring system was predictive of mortality. While we have previously evaluated and utilized this score in rat models of sepsis [5, 41], this is the first description of its use in a murine model, where mortality and serum biochemical markers of organ failure correlated with clinical severity (data not shown). All mice that scored ≥4 subsequently died, while those with scores ≤3 survived.

The severely septic mice developed a mixed respiratory and metabolic acidosis with hyperlactataemia. Hyperchloraemia also contributes in part to the metabolic acidosis; as this was also observed in the sham mice, it is likely related to the chloride-rich fluid used for fluid resuscitation, which may have been avoided had a balanced crystalloid solution been used instead. Nevertheless, acid–base balance was relatively unaffected in the severely septic rats with only mild hypocapnia and hyperlactataemia.

Limitations of our study include an inability to directly reproduce the study design in both rodent species. We did initially use a tether system and vascular instrumentation under anaesthesia to enable continuous intravenous fluid administration in mice, but this proved too stressful and injurious, even for non-septic controls, and had to be abandoned. Thus, the mice did not receive any surgical trauma and their fluid regimens were, by necessity, different. Importantly, however, the 72-h mortality rates were identical so we opted not to use a model involving repeated subcutaneous fluid injections in the rats. We also did not use antibiotics as the intention was to compare haemodynamic and metabolic responses between species with an insult of comparable mortality.

## Conclusions

We demonstrate markedly differing metabolic and cardiovascular profiles in long-term rat and mouse models of bacterial sepsis with similar mortality. The mouse model, in particular, exhibits early severity-dependent metabolic down-regulation soon after the initiation of sepsis. Other models of sepsis involving different insults, treatment regimens, laboratory conditions (e.g. ambient temperatures), ages and gender should also be examined to assess reproducibility of findings. In the meantime, we suggest that caution be applied in extrapolating findings in murine models to septic patients, both with regard to our understanding of pathophysiology and the failure to translate preclinical efficacy into successful clinical trials.

## Electronic supplementary material

Additional file 1: Figure S1: Averaged trace of VO_2_ of two naïve mice and four naïve rats over 48 to 72 h with 12-h dark periods marked (grey bar at top). Error bars depict standard deviation. (PPTX 55 KB)
