# Supplementary figures and images for: The metabolic phenotype of rodent sepsis: cause for concern?
Source: Intensive Care Med Exp. 2013 Oct 29;1:6. doi: 10.1186/2197-425X-1-6 (PMC4797805; doi:10.1186/2197-425X-1-6)

## Slide 1
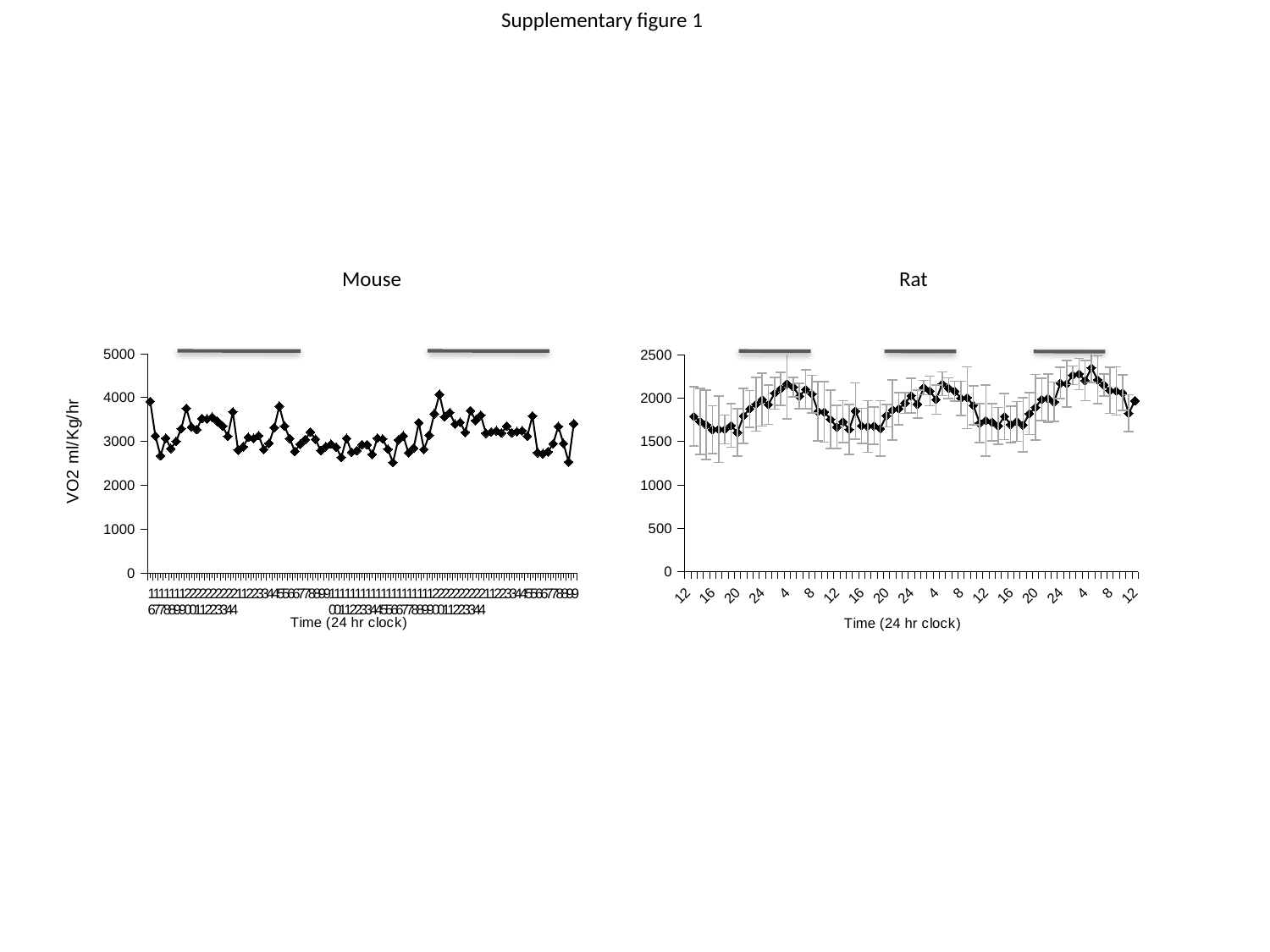

Supplementary figure 1
Mouse
Rat
### Chart
| Category | |
|---|---|
| 12 | None |
| 13 | 1789.74192389225 |
| 14 | 1732.0967711080107 |
| 15 | 1693.209299268024 |
| 16 | 1637.10065626685 |
| 17 | 1642.016435108245 |
| 18 | 1639.3857231491957 |
| 19 | 1684.042756874737 |
| 20 | 1604.548989193941 |
| 21 | 1795.404571339936 |
| 22 | 1875.89157600065 |
| 23 | 1930.2599115867847 |
| 24 | 1983.7753694040057 |
| 1 | 1926.30989626035 |
| 2 | 2057.687217753551 |
| 3 | 2108.765070870911 |
| 4 | 2165.315210470726 |
| 5 | 2128.1686539327998 |
| 6 | 2023.34717402705 |
| 7 | 2100.8845487380618 |
| 8 | 2047.6584838278413 |
| 9 | 1847.783800143293 |
| 10 | 1841.0013409706985 |
| 11 | 1756.60607628502 |
| 12 | 1667.5823923271803 |
| 13 | 1729.0247797055856 |
| 14 | 1639.02942564648 |
| 15 | 1852.710520827947 |
| 16 | 1681.9739956568949 |
| 17 | 1675.067856635054 |
| 18 | 1680.847788654007 |
| 19 | 1649.82517930082 |
| 20 | 1798.5784702371714 |
| 21 | 1864.694587368064 |
| 22 | 1877.6314025718857 |
| 23 | 1943.819102646487 |
| 24 | 2029.069273268482 |
| 1 | 1932.1947029626178 |
| 2 | 2120.8640345823464 |
| 3 | 2082.308178776397 |
| 4 | 1983.614601106458 |
| 5 | 2164.058102698192 |
| 6 | 2114.9907372938333 |
| 7 | 2079.337689733874 |
| 8 | 1997.9459877979427 |
| 9 | 2005.832493149268 |
| 10 | 1916.524301677783 |
| 11 | 1712.573821341783 |
| 12 | 1741.9692706833 |
| 13 | 1724.3930004357878 |
| 14 | 1681.8269100184402 |
| 15 | 1789.1672427928822 |
| 16 | 1697.2724046621909 |
| 17 | 1731.782213949641 |
| 18 | 1690.71098517583 |
| 19 | 1821.817906076654 |
| 20 | 1894.6890945954922 |
| 21 | 1986.027527384461 |
| 22 | 1999.0586540805257 |
| 23 | 1956.52337558259 |
| 24 | 2174.1864964878437 |
| 1 | 2164.9230805025613 |
| 2 | 2264.69651591364 |
| 3 | 2279.042857973808 |
| 4 | 2202.5202313553 |
| 5 | 2349.353559795255 |
| 6 | 2211.1840202530507 |
| 7 | 2151.7797860946757 |
| 8 | 2088.959728408194 |
| 9 | 2083.387002075533 |
| 10 | 2061.153903624426 |
| 11 | 1824.706414205204 |
| 12 | 1969.705232407838 |
### Chart
| Category | |
|---|---|
| 16 | 3909.0 |
| 16.5 | 3120.625 |
| 17 | 2669.25 |
| 17.5 | 3068.75 |
| 18 | 2834.5 |
| 18.5 | 2997.125 |
| 19 | 3284.5 |
| 19.5 | 3751.25 |
| 20 | 3326.125 |
| 20.5 | 3267.75 |
| 21 | 3518.125 |
| 21.5 | 3507.625 |
| 22 | 3547.8750000000014 |
| 22.5 | 3454.125 |
| 23 | 3350.625 |
| 23.5 | 3111.8750000000014 |
| 24 | 3669.25 |
| 0.5 | 2797.125 |
| 1 | 2873.75 |
| 1.5 | 3088.75 |
| 2 | 3063.625 |
| 2.5 | 3128.125 |
| 3 | 2813.625 |
| 3.5 | 2952.625 |
| 4 | 3311.0 |
| 4.5 | 3798.25 |
| 5 | 3346.625 |
| 5.5 | 3062.25 |
| 6 | 2767.75 |
| 6.5 | 2934.3750000000014 |
| 7 | 3040.5 |
| 7.5 | 3210.3750000000014 |
| 8 | 3045.125 |
| 8.5 | 2787.0 |
| 9 | 2876.3750000000014 |
| 9.5 | 2935.75 |
| 10 | 2867.5 |
| 10.5 | 2631.5 |
| 11 | 3065.625 |
| 11.5 | 2750.5 |
| 12 | 2784.625 |
| 12.5 | 2921.25 |
| 13 | 2920.5 |
| 13.5 | 2700.8750000000014 |
| 14 | 3069.3750000000014 |
| 14.5 | 3052.5 |
| 15 | 2818.75 |
| 15.5 | 2517.3750000000014 |
| 16 | 3027.625 |
| 16.5 | 3122.75 |
| 17 | 2733.25 |
| 17.5 | 2841.75 |
| 18 | 3424.625 |
| 18.5 | 2814.125 |
| 19 | 3137.125 |
| 19.5 | 3624.625 |
| 20 | 4067.25 |
| 20.5 | 3556.5 |
| 21 | 3654.125 |
| 21.5 | 3391.5 |
| 22 | 3432.75 |
| 22.5 | 3203.0 |
| 23 | 3698.25 |
| 23.5 | 3472.125 |
| 24 | 3590.75 |
| 0.5 | 3182.8750000000014 |
| 1 | 3209.5 |
| 1.5 | 3238.0 |
| 2 | 3188.25 |
| 2.5 | 3344.625 |
| 3 | 3193.0 |
| 3.5 | 3218.75 |
| 4 | 3239.3750000000014 |
| 4.5 | 3114.5 |
| 5 | 3578.0 |
| 5.5 | 2741.0 |
| 6 | 2715.0 |
| 6.5 | 2759.125 |
| 7 | 2944.0 |
| 7.5 | 3337.75 |
| 8 | 2949.125 |
| 8.5 | 2527.5 |
| 9 | 3402.0 |

Supplement: Supplementary file 1 — Additional file 1: Figure S1: Averaged trace of VO2 of two naïve mice and four naïve rats over 48 to 72 h with 12-h dark periods marked (grey bar at top). Error bars depict standard deviation. (PPTX 55 KB) [file 40635_2013_25_MOESM1_ESM.pptx]
